# Can policy participation behavior improve the production efficiency of grassland and animal husbandry: A test based on the threshold effect perceived by herdsmen

**DOI:** 10.1371/journal.pone.0339538

**Published:** 2026-02-26

**Authors:** Xin Zhang, Xiaolong Yuan, Haiying Liu

**Affiliations:** Department of Management, Ordos Institute of Technology, Ordos, Inner Mongolia Autonomous Region, China; PLOS ONE, UNITED KINGDOM OF GREAT BRITAIN AND NORTHERN IRELAND

## Abstract

Based on survey data from 536 pastoral households in Inner Mongolia, this study employs the Propensity Score Matching (PSM) method to estimate the Average Treatment Effect on the Treated (ATT) of herders’ participation in grassland ecological compensation policies on their grass and animal husbandry production efficiency during the first and second policy rounds. Furthermore, we utilize herders’ perceptions as a threshold variable to investigate the nonlinear effects of policy participation on production efficiency. The results indicate that: (1) Policy participation significantly suppressed production efficiency during the first policy period, while its effect in the second period was statistically insignificant. (2) Threshold regression models reveal a nonlinear, U-shaped relationship between policy participation and production efficiency, dependent on herders’ overall, economic, and loss perceptions. Specifically, significant double-threshold effects were identified for these three perception types. While overall and loss perceptions remain in the significant inhibitory phase at the lower threshold range, economic perception has already surpassed its lower threshold, enabling policy participation to effectively promote production efficiency. In contrast, only single-threshold effects were found for ecological and emotional perceptions, with policy participation showing no significant positive effect even at higher perception levels.

## Introduction

The 19th National Congress of the Communist Party of China proposed the major strategic decision to implement the Rural Revitalization Strategy, emphasizing that issues relating to agriculture, rural areas, and farmers are fundamental to national development and people’s livelihoods. Addressing these “San Nong” issues must remain a top priority, with the implementation of the Rural Revitalization Strategy being central to this effort. Pastoral areas hold significant strategic importance in China’s overall economic and social development; thus, revitalizing these regions is essential for achieving the goal of building a modern socialist country [1]. As a renewable resource, grassland plays a crucial role in developing grass-based livestock production, protecting biodiversity, and maintaining ecological balance [2,3]. China, with its vast grassland resources, possesses approximately 400 million hectares of various natural grasslands, covering nearly two-fifths of the country’s land area [4]. Most pastoral areas in China are located in arid and semi-arid climates, where grassland ecosystems are highly fragile. These regions have long faced the challenge of balancing economic production in animal husbandry with the protection of ecological values [5]. In response to the severe degradation of high-quality forage and the deterioration of grassland vegetation structure [6], the Chinese government initiated a comprehensive grassland ecological protection subsidy and reward mechanism in 2011. In 2016, the government further introduced the “Guidance on Implementing a New Round of Grassland ecological compensation policy,” emphasizing efforts to enhance grazing exclusion and grassland restoration while increasing compensation standards. As one of the most critical support policies for pastoral area development, Grassland ecological compensation policy represents the largest investment, most extensive coverage, and broadest involvement of farmers and pastoralists in China’s pastoral regions [7]. The policy encourages herders to reduce livestock numbers and protect the grassland ecological environment through measures such as grazing bans and grass-animal balance systems. Research on the Grassland ecological compensation policy has yielded valuable insights, ranging from policy mechanism design to the evaluation of implementation outcomes [8,9]. A key focus among scholars has been examining the trade-offs between changes in individual herder households and the achievement of policy objectives, with studies covering aspects such as herder income and welfare, satisfaction levels, and livestock scale [10–12].

The “Opinions on Doing a Good Job in Key Tasks for Comprehensively Advancing Rural Revitalization in 2022” emphasizes strengthening the fundamental support for modern agriculture. As a foundational industry in the pastoral economy, grass-based animal husbandry remains irreplaceable in promoting sustainable development in pastoral areas and improving the quality of life for herders [13]. However, grassland degradation severely constrains the sustainable development of both production and livelihoods in these regions. Therefore, it is essential to objectively assess the development level of grass-based animal husbandry in pastoral areas and explore ways to achieve sustainable production improvements while simultaneously protecting the grassland ecosystem. The second phase of the Grassland ecological compensation policy (2016–2020) has concluded. Beyond substantial government investment, the protection of the grassland ecological environment requires active participation and effective supervision from herders [14]. Since the policy’s implementation, varying levels of herders’ perception and understanding have been a significant factor contributing to its suboptimal outcomes. With socio-economic development, disparities in perception have become increasingly pronounced, and herders’ subjective perceptions of the policy play a crucial role in determining its effectiveness. Self-management behaviors derived from herders’ subjective perceptions can not only mitigate the high costs associated with policy supervision and enforcement in grassland ecological protection but also foster voluntary actions to safeguard grassland ecosystems, thereby ensuring the achievement of the policy’s objectives [15].

Against this backdrop, this study examines the relationship between herders’ participation in the Grassland ecological compensation policy and the efficiency of their grass and animal husbandry production. From the perspective of herders’ perceptions, it investigates the threshold effects of policy participation on production efficiency. The findings are expected to provide valuable insights for improving herders’ production efficiency, refining the next phase of the grassland ecological compensation mechanism, effectively promoting green industrial development in pastoral areas, and ultimately contributing to the revitalization of these regions.

## Materials and methods

### Data sources

The data involved in this paper comes from the field investigation of herdsmen engaged in animal husbandry production before and after the implementation of the first and second rounds of grassland ecological compensation policies in Inner Mongolia Autonomous Region, including 2010, 2015 and 2020. In order to maintain the consistency of the samples, the pure animal husbandry counties in Inner Mongolia were selected as the survey areas. The selection of study areas in this research followed the principles of representativeness, typicality, and feasibility. The eight pure pastoral banners selected—Siziwang Banner, Jarud Banner, Chen Barag Banner, New Barag Left Banner, Abag Banner, Sonid Right Banner, Hexigten Banner, and Bairin Right Banner—demonstrate strong representativeness in terms of geographical distribution, grassland types, and socio-economic conditions, effectively reflecting the overall situation of Inner Mongolia’s pastoral regions. These sample banners span approximately 1,600 kilometers from east to west, covering major grassland areas from the Hulun Buir Plateau in eastern Inner Mongolia, through the Xilingol Plateau in the central region, to the Horqin Sandy Land and Ulanqab Plateau in the south. This extensive coverage ensures that the study includes the most representative ecological types of Inner Mongolia’s grasslands: meadow steppes (e.g., Chen Barag Banner), typical steppes (e.g., Abag Banner), and desert steppes (e.g., Sonid Right Banner, Siziwang Banner). Consequently, the research captures the heterogeneity of policy implementation effects under different hydrothermal conditions and ecological backgrounds. All selected banners are core and key implementation areas for the national and Inner Mongolia autonomous region’s Grassland ecological compensation policy. In these regions, animal husbandry is the dominant industry, and the conflict between grassland ecological protection and herders’ livelihoods is most acute. Therefore, studying these areas provides the most revealing insights into the micro-level effects of policy implementation, and the conclusions offer direct reference value for optimizing ecological compensation policies across the entire grassland pastoral region. In summary, Based on the actual development of each county and the distribution of grassland types, this survey adopts a combination of random sampling and typical sampling for one-to-one access data collection. The survey covers 5 cities, 8 pure animal husbandry counties and 23 towns including Hulunbuir City, Xilin Gol League, Chifeng City, Tongliao City and Ulanqab city in Inner Mongolia. Excluding the samples with missing important indicators, there are 536 valid questionnaires. The sample distribution is shown in Table 1. Prior to each interview, researchers clearly identified themselves as faculty and graduate students from the Ordos Institute of Technology. Participants were verbally informed about: the research focus on grassland ecological compensation policies and household livestock production, the academic purpose of data collection, strict confidentiality measures, and their right to decline or discontinue participation. The agreement was obtained before proceeding with the survey. Our methodology aligns with standard practices in agricultural economics research where surveys collect production data and policy perceptions without engaging in human subjects research as ethically defined. All participants received transparent information about the research purpose and data handling procedures.

**Table 1:** Sample distribution in the survey area.

League or city	County or banner	League or city sample size	Sample size	Percentage of sample (%)
**Hulunbuir**	Chenbarhu Banner	139	62	11.57
	Xin Barag Left Banner		77	14.36
**Xilin Gol**	Abaga Banner	135	78	14.55
	Sonid Right Banner		57	10.63
**Chifeng**	Hexigten Banner	114	53	9.88
	Balin Right Banner		61	11.38
**Ulanqab**	Siziwang Banner	69	69	12.88
**Tongliao**	Jarud Banner	79	79	14.74
**Total**	–	536	536	100

### Methods

#### Propensity score matching method.

This paper uses the propensity score matching method to analyze the impact of herdsmen’s policy participation behavior on their grass and animal husbandry production efficiency during the first and second round of grassland ecological compensation policy. Whether herdsmen participate in the policy is not a random distribution, but a self-selection problem made by herdsmen according to their own subjective and objective factors. The propensity score matching rule is one of the main methods to deal with the self-selection problem. In this paper, the herdsmen without policy participation behavior were selected as the control group, and the treatment group and the control group were set up. It is used to analyze whether there is a significant difference in the production efficiency of grass and animal husbandry between herdsmen who participate in the policy and those who do not participate, that is, whether the average treatment effect is significant. The virtual variable Di={0,1} is used to indicate whether herdsman I has policy participation behavior, that is, “1” means there is policy participation behavior, and “0” means there is no policy participation behavior. Y_i_ is the result of grass and animal husbandry production efficiency. For individual herdsmen i, Y_i_ may have two states, depending on whether herdsmen participate in the policy [16].


$$Y_{i}=\left\{ \begin{array}{c} \;\;\;Y_{i}^{\text{1}}\;D_{i}=\text{1}\\ \;\;\;Y_{i}^{\text{0}}\;D_{i}=\text{0} \end{array}\right.$$ (1)

where, Y_i_^0^ represents the production efficiency of grass and animal husbandry without the participation of herdsmen i in the policy, and Y_i_^1^ represents the result after the participation of herdsmen I in the policy. Y_i_ can be written as:


$$Y_{i}=\left(\text{1}-D_{i}\right)Y_{i}^{\text{0}}+D_{i}Y_{i}^{\text{1}}=Y_{i}^{\text{0}}+\underset{\text{Treatment effect}}{\underbrace{\left(Y_{i}^{\text{1}}-Y_{i}^{\text{0}}\right)}}D_{i}$$ (2)

Y_i_^1^-Y_i_^0^ refers to the treatment effect of policy participation behavior of individual of herdsman. The average treatment effect is calculated according to the matched herdsman samples. The general expression of the average treatment effect estimator of herdsman with policy participation behavior is as follows, where N_1_ is the number of individuals in the treatment group.


$$ATT=\frac{\text{1}}{N_{\text{1}}}\sum_{D_{i}=\text{1}}\left(Y_{i}-Y_{i}^{\text{0}}\right)$$ (3)

#### Threshold effect estimation method.

Threshold effect refers to the phenomenon that when one economic parameter reaches a specific value, another economic parameter suddenly turns to other forms of development (structural mutation). The critical value of the causal phenomenon is called threshold value. Threshold regression proposed by Hansen uses a strict statistical inference method to estimate the parameters and test the hypothesis of the threshold [17]. The threshold effect regression model is as follows:


$$y_{it}=\mu_{i}+\beta_{\text{2}}^{\prime }x_{it}+\varepsilon_{it},\;if\;q_{it}\geq \gamma$$ (4)

$$y_{it}=\mu_{i}+\beta_{\text{1}}^{\prime }x_{it}+\varepsilon_{it},\;if\;q_{it}<\gamma$$ (5)

It can also be expressed as:


$$y_{it}=\mu_{i}+\beta_{\text{1}}^{\prime }x_{it}\times I(q_{it}\geq \gamma )+\beta_{\text{2}}^{\prime }x_{it}\times I(q_{it}<\gamma )+\varepsilon_{it}$$ (6)

where, *i* in the above formula refers to each region, *t* refers to each time year. *q*_*it*_ is the threshold variable, *γ* is the threshold value, and the sample data of the threshold variable is divided into different intervals. *I*() is an indicative function. If the threshold variable in the bracket meets the conditions, the indicator function is taken as 1; otherwise, it is taken as 0. $\beta_{\text{1}}^{\prime }$ and $\beta_{\text{2}}^{\prime }$ is the parameter to be estimated, *ε*_*it*_ is the random disturbance term, which obeys the independent identically distributed.

Referring to Hansen (1997)‘s research on threshold effect, this paper analyzes the threshold effect of herdsmen’s participation in the policy on their grass and animal husbandry production efficiency under the grassland ecological compensation policy by taking herdsmen’s subjective perception level, economic perception, ecological perception, loss perception and emotional perception as threshold variables. First, set the model as a single threshold model, as shown in models (7) - (10).


$${TFP}_{it}=\beta_{\text{0}}+\beta_{\text{1}}{treat}_{it}*I\left({perception}_{it}\geq \gamma \right)+\beta_{\text{2}}{treat}_{it}*I\left({perception}_{it}<\gamma \right)+\beta_{\text{3}}*{CV}_{it}+\varepsilon$$ (7)

$${TFP}_{it}=\beta_{\text{0}}+\beta_{\text{1}}{treat}_{it}*I\left({economy}_{it}\geq \gamma \right)+\beta_{\text{2}}{treat}_{it}*I\left({economy}_{it}<\gamma \right)+\beta_{\text{3}}*{CV}_{it}+\varepsilon$$ (8)

$${TFP}_{it}=\beta_{\text{0}}+\beta_{\text{1}}{treat}_{it}*I\left({loss}_{it}\geq \gamma \right)+\beta_{\text{2}}{treat}_{it}*I\left({loss}_{it}<\gamma \right)+\beta_{\text{3}}*{CV}_{it}+\varepsilon$$ (9)

$${TFP}_{it}=\beta_{\text{0}}+\beta_{\text{1}}{treat}_{it}*I\left({emotion}_{it}\geq \gamma \right)+\beta_{\text{2}}{treat}_{it}*I\left({emotion}_{it}<\gamma \right)+\beta_{\text{3}}*{CV}_{it}+\varepsilon$$ (10)

$${TFP}_{it}=\beta_{\text{0}}+\beta_{\text{1}}{treat}_{it}*I\left({eco\text{ log }y}_{it}\geq \gamma \right)+\beta_{\text{2}}{treat}_{it}*I\left({eco\text{ log }y}_{it}<\gamma \right)+\beta_{\text{3}}*{CV}_{it}+\varepsilon$$ (11)

where, i refers to individual herdsmen, t is the time. TFP_it_ indicates the production efficiency of herdsmen’s grass husbandry. Treat_it_ indicates Whether there is policy participation. CV_it_ represents the corresponding control variable. *I*(·) represents an indicative function. perceptionit, economyit, lossit, emotionit and ecologyit are threshold variables, which respectively represent the overall perception, economic perception, loss perception, emotional perception and Ecological Perception of herdsmen. γ Indicates the threshold value to be evaluated.

### Variable selection

#### Dependent variable.

In this paper, the production efficiency of herdsmen’s grass husbandry is taken as the dependent variable. The methods used by academia in the process of studying production efficiency and technical efficiency are mainly divided into two categories: parametric estimation method and nonparametric estimation method [18]. Compared with the parameter estimation method, the basic distribution of the nonparametric estimation method is not assumed, and DEA Malmquist index is widely used [19]. Therefore, this paper uses DEA Malmquist index method to measure the total factor productivity to measure the production efficiency of grass and animal husbandry. In measuring the total factor productivity of herdsmen’s grass and animal husbandry, the selection of input and output indicators in this paper follows common academic practices while closely integrating the production characteristics of grassland animal husbandry and research objectives. The DEA model requires that input and output indicators comprehensively and non-redundantly reflect resource consumption and final outcomes in the production process. Accordingly, the input indicators aim to cover all essential core production factors for herdsmen’s grass and animal husbandry operations, including land (actual grassland management area), labor (household labor input), capital (livestock inventory representing biological asset stock), and variable cash costs (production cost inputs). This combination ensures comprehensive capture of production inputs from both static (asset) and dynamic (cash flow) perspectives.

The selection of output indicators focuses on the ultimate economic and physical outcomes of herdsmen’s grass and animal husbandry operations. The number of slaughtered livestock represents core physical products created through the production process that can be directly marketed or used for household consumption, while total operational income from grass and animal husbandry comprehensively reflects the market realization degree of all outputs (including both primary products and by-products).

In terms of the selection of input and output indicators for measuring the total factor productivity of herdsmen’s Grassland and animal husbandry, this paper takes the actual operating grassland area, labor input, the number of livestock on hand, and the production cost input of grassland and animal husbandry as the input indicators for herdsmen’s Grassland and animal husbandry production, and the number of livestock and the total operating income of grassland and animal husbandry as the main output indicators [20,21]. Among them, grassland input, as an important means of production for herdsmen’s grass and animal husbandry production, provides the basis for the allocation of other production factors [22]. The input of grassland actual operating area specifically includes the sum of the grassland area owned, used and rented by herdsmen ‘families, minus the grassland area rented out. Labor input specifically refers to the number of people engaged in livestock breeding in the family. The number of livestock on hand refers to the number of livestock on hand at the beginning of the year, mainly including the sum of the number of cattle (beef cattle, dairy cows, etc.), sheep (sheep, goats, etc.) and horses, which are converted according to the sheep unit. The cost input of forage and animal husbandry mainly includes the input cost of forage, the cost of grassland lease, the cost of employees, medical expenses, grazing expenses and fuel costs, among which the input cost of forage mainly includes the cost of forage and feed salt purchase, the cost of artificial grassland feeding, etc. Labor costs mainly include labor and machinery costs for mowing (mowing, bundling and pulling grass, etc.), labor costs for sheep washing and wool shearing, etc. Medical expenses mainly include medical expenses for sheep and cattle and epidemic prevention expenses. The number of livestock for slaughter mainly includes cattle (beef cattle, dairy cows, etc.), sheep (sheep, goats, etc.) and horses and other livestock converted by sheep unit. The total operating income of grass and animal husbandry mainly includes the income from livestock slaughter, the income from grassland rental, the income from the sale of pasture and the income from wool, sheepskin, milk and other by-products. The number of livestock is converted in accordance with the sheep unit. The conversion standard is: 2 piglets = 1 adult animal [23], 1 sheep = 1 sheep unit, 1 cow = 5 sheep units, and 1 horse = 6 sheep units (The conversion shall be carried out according to the conversion method of sheep unit in the supplementary provisions of the regulations of Inner Mongolia Autonomous Region on the protection of basic grasslands.). See Table 2 for the explanation and value of specific input-output indicators of herdsmen.

**Table 2:** Input output index selection.

Type	Variable	Variable interpretation and value	Unit
**Output indicators**	The number of livestock sold	The number of cattle, sheep, horses and other livestock to be sold in sheep units.	Sheep unit
	The total operating income of animal husbandry	The income from livestock sales, grassland rental, grass sales, wool, sheepskin, milk and other by-products.	10000RMB
**Input indicators**	Grassland actual operating area	Actual contracted grassland area + leased grassland area -rented grassland area	Hectare
	Labor input	The number of herdsmen engaged in livestock breeding	Person
	The number of livestock on hand	The number of cattle, sheep, horses and other livestock on hand in sheep units.	Sheep unit
	The cost input of animal husbandry	The cost of forage, the cost of grassland lease, the cost of employment, the cost of medicine, the cost of grazing and the cost of fuel oil, etc.	10000RMB

#### Core independent variables and control variables.

In this paper, whether there is policy participation behavior of herdsmen during the implementation of the first round and the second round of grassland ecological compensation policy is taken as the core independent variable to test the effect on the production efficiency of herdsmen’s grass husbandry. Herders’ policy participation behavior refers to the process and state in which herders, in response to the incentive and constraint mechanisms of the Grassland Ecological Compensation Policy, actively or passively adjust their grass and animal husbandry production practices to align with the policy’s “grass-livestock balance” objective. To accurately identify policy participants and minimize subjective judgment bias, this study adopts the following operational definitions for participation behavior across different policy cycles:

During the first phase of the compensation policy, which primarily focused on guiding and encouraging livestock reduction, any herder who reduced their livestock inventory within this period was defined as an actual policy participant. As the policy evolved with stricter supervision during the second phase, mere livestock reduction became insufficient to reflect sustained compliance. Therefore, this study applied objective compliance criteria for determination. Specifically, based on the reasonable stocking capacity standards for grasslands published by local animal husbandry and grassland authorities, herders whose average annual livestock inventory consistently met or remained below this standard throughout the policy period were identified as actual participants. This approach emphasizes behavioral continuity and outcome compliance, aiming to more precisely measure the production behavior transformation desired by the policy.

Before and after the implementation of the first round of policies (2010–2015), herdsmen who participated in the policies were regarded as the treatment group of the first round of policies, and herdsmen who did not participate in the policies were regarded as the control group for propensity score matching. Among them, 43.84% of herdsmen had policy participation in the first round of policies, and 56.16% of herdsmen had no policy participation (see Table 3).When identifying the policy participation behavior of herdsmen before and after the implementation of the second round of policies (2015–2020), the herdsmen who strictly reduced their livestock to the range of livestock carrying capacity specified in the policy on the basis of the first round of policies were regarded as the treatment group of the second round of supplementary award policy. On the contrary, it was the control group, in which 29.66% of herdsmen reduced their livestock to the range of livestock carrying capacity specified in the policy, and 70.34% of herdsmen did not reduce their livestock to the range of livestock carrying capacity specified in the policy.

**Table 3:** Variable definition and value range.

Type		Variable	Variable interpretation and value	Unit/ Assignment	Frequency (%)	Mean	Standard deviation
Core independent variables	Livestock reduction behavior of herdsmen	Livestock reduction during the first round of policy	Whether herdsmen have reduced livestock.	No =0	56.16	0.44	0.50
				Yes =1	43.84		
		Livestock reduction during the second round of policy	Whether herdsmen reduce livestock in strict accordance with the policy.	No =0	70.34	0.30	0.46
				Yes =1	29.66		
Control variable	Personal characteristics of herdsmen	Gender	Female =0, Male =1	Female =0	6.95	0.93	0.25
				Male =1	93.05		
		Age	Age of head of household	Year	-	47.79	10.66
		Education level	1= Primary school and below, 2= Junior high school, 3= Senior high school, 4= Technical secondary school or college, 5= Undergraduate and above	Primary school and below =1	26.01	2.14	1.11
				Junior high school =2	50.89		
				Senior high school =3	15.02		
				Technical secondary school or college =4	4.85		
				Undergraduate and above =5	3.23		
	Family characteristics	Whether to work part-time	Whether there are other stable incomes other than animal husbandry.	No =0	73.43	0.27	0.44
				Yes =1	26.57		
		Family dependency ratio	Number of dependents / Total family population	%	-	0.39	0.24
	Traffic conditions	Distance to the market	The distance from the family to the nearest market.	Km	-	27.70	57.60
		Travel convenience	Whether there is a vehicle.	No =0	32.96	0.67	0.47
				Yes =1	67.04		
	Economic conditions	Compensation amount	Compensation standard × Grassland contracted area	10000RMB	-	2.22	2.37
		Yearly consumption expenditure	Yearly consumption expenditure of clothing, food, housing and transportation	10000RMB	-	4.35	3.43
	Supervision strength	Government supervision strength	Whether the government has supervised the animal husbandry production of herdsmen.	None at all =1	15.19	2.51	0.93
				Yes, rarely =2	33.76		
				Some =3	37.00		
				Yes, more =4	13.25		
				Yes, very strict =5	0.81		
		Whether to be fined	Are herdsmen fined for overloading.	No =0	65.67	0.34	0.47
				Yes =1	34.33		

The common control variables are selected from five aspects of herdsmen’s personal characteristics, family characteristics, traffic conditions, economic conditions and supervision [24,25], including gender, age, education level, whether to work part-time, family dependency ratio, distance to the market, travel convenience, compensation amount, yearly consumption expenditure, government supervision and whether to be fined. It can be seen from Table 3 that in terms of personal and family characteristics, the average age of the head of household is 47.79, male accounts for a relatively high proportion, and the average family dependency ratio is 0.39. The overall education level of the surveyed herdsmen is mainly concentrated in primary school and junior high school education, accounting for 76.9%, and the overall education level is low. Herders relying solely on grass and animal husbandry production as their income source account for 73.43% of the total sample, while less than 30% of herders have supplementary income from diversified operations. This indicates that grass and animal husbandry production remains the primary source of stable household income for the majority of herders. From the aspect of traffic conditions, it can be seen that the average household is 27.7 km away from the nearest market. The distance is moderate, and 67.04% of herdsmen’s households have vehicles. The traffic conditions are convenient, but the degree of dispersion is high, and the gap between herdsmen’s households is large. Regarding economic conditions, the average household compensation amount was 22,200 CNY, though with considerable dispersion, indicating substantial variation in compensation sums among herders. In terms of regulatory intensity, 34.33% of herders reported receiving fines for overloading, while 84.82% perceived some form of government supervision. The mean perception score of 2.51 falls between “rarely present” and “moderately present,” suggesting an overall low-to-moderate level of regulatory enforcement.

#### Threshold variable.

We recognize that relying solely on binary classifications such as “livestock reduction yes/no” or “compliance yes/no” may not fully capture the heterogeneity in herder participation. For instance, this approach fails to distinguish between herders who proactively adjust their practices based on a thorough understanding of policy objectives and those who passively comply due to external pressure. The potential behavioral motivation bias arising from such differences in subjective cognition constitutes a key focus of this study. To address this limitation, we introduce “herdsman perception” as a threshold variable to examine the differential impacts of policy participation on production efficiency across varying levels of perception. This methodological approach not only enables a more nuanced revelation of the intrinsic mechanisms through which the policy operates but also provides an effective analytical tool for identifying and discerning potential subjective judgment biases.

This paper constructs the subjective perception level of herdsmen ‘families on grassland ecological compensation policy from four dimensions, including economic perception, loss perception, ecological perception and emotional perception. This paper constructs the subjective perception level of herdsmen ‘families on grassland ecological compensation policy from four dimensions, including economic perception, loss perception, ecological perception and emotional perception. Among them, the paper selects “whether the implementation of the policy has improved the overall income level of your family” and “the degree of improvement of the policy on your production and life” as the observable variables of economic perception. The loss perception is marked by “whether you think the existing compensation standard can make up for the loss caused by the implementation of the compensation mechanism” and “whether you think the implementation of the compensation policy has reduced the input cost”. The ecological perception is represented by “changes in the height and density of pastures after the policy” and “changes in the overall ecological environment of pastures in your opinion”. And use “do you think the policy has promoted the overall progress of pastoral society” and “do you think your dependence on the policy” as observable variables of emotional perception. In this study, the mean values of observed variables are used to represent herdsmen’s economic perception, ecological perception, loss perception, and emotional perception toward the compensation policy. To measure herdsmen’s multidimensional perceptions of the grassland ecological compensation policy more scientifically and objectively, the entropy method is employed to construct a comprehensive index of overall perception. The entropy method is an objective weighting technique that determines weights based on the amount of information provided by each observed indicator, effectively avoiding subjective arbitrariness. The entropy method was employed to determine the weights of the four perception dimensions, yielding the following results: economic perception (0.247), ecological perception (0.256), loss perception (0.251), and emotional perception (0.246). The relatively balanced distribution of weights across the dimensions indicates that each contributes similarly to the comprehensive perception index, with ecological perception carrying slightly greater importance. This weighting scheme effectively captures the multidimensional nature of herdsmen’s perceptions while minimizing subjective bias in the aggregation process. Table 4 reports the mean value and standard deviation of threshold variables. It can be seen that the mean value of herdsmen’s perception of the policy is mainly concentrated between “2” and “3”. On the whole, herdsmen’s perception of the subsidy policy is still at a lower medium level.

**Table 4:** Threshold variable definition and value range.

Type	Variable	Variable value range	Mean	Standard deviation
**Overall perception of herdsmen**	The overall perception level of herdsman families on policy.	Comprehensive values of economic perception, ecological perception, loss perception, and emotional perception	2.497	0.954
**Economic perception**	Whether the implementation of the policy has improved the overall income level of your family.	1 = significantly worse, 2 = slightly worse, 3 = no change, 4 = slightly higher, 5 = significantly higher	2.488	0.933
	The degree to which the policy has improved your production and life.	1 = significantly worse, 2 = slightly worse, 3 = no change, 4 = slightly better, 5 = significantly better	2.499	0.969
**Ecological perception**	Changes in the height and density of pastures after the policy.	11 = significantly worse, 2 = slightly worse, 3 = no change, 4 = slightly better, 5 = significantly better	2.574	0.99
	Changes in the overall ecological environment of pastures in your opinion.	1 = significantly worse, 2 = slightly worse, 3 = no change, 4 = slightly better, 5 = significantly better	2.483	0.935
**Loss perception**	Whether you think the existing compensation standard can make up for the loss caused by the implementation of the compensation mechanism.	1 = cannot be compensated at all, 2 = partially compensated, 3 = basically compensated, 4 = small amount of compensated excess, 5 = large amount of compensated excess	2.482	0.962
	Whether you think the implementation of the compensation policy has reduced the input cost.	1 = substantial increase, 2 = increase a little, 3 = no change, 4 = decrease a little, 5 = substantial decrease	2.494	0.94
**Emotional perception**	Do you think the policy has promoted the overall progress of pastoral society.	1 = significantly worse, 2 = slightly worse, 3 = no change, 4 = slightly better, 5 = significantly better	2.467	0.945
	Do you think your dependence on the policy.	1 = completely independent, 2 = relatively independent, 3 = dependent, 4 = relatively dependent, 5 = completely dependent	2.490	0.957

## Results

### Analysis of production efficiency in pastoral grassland animal husbandry

**Fig 1:**
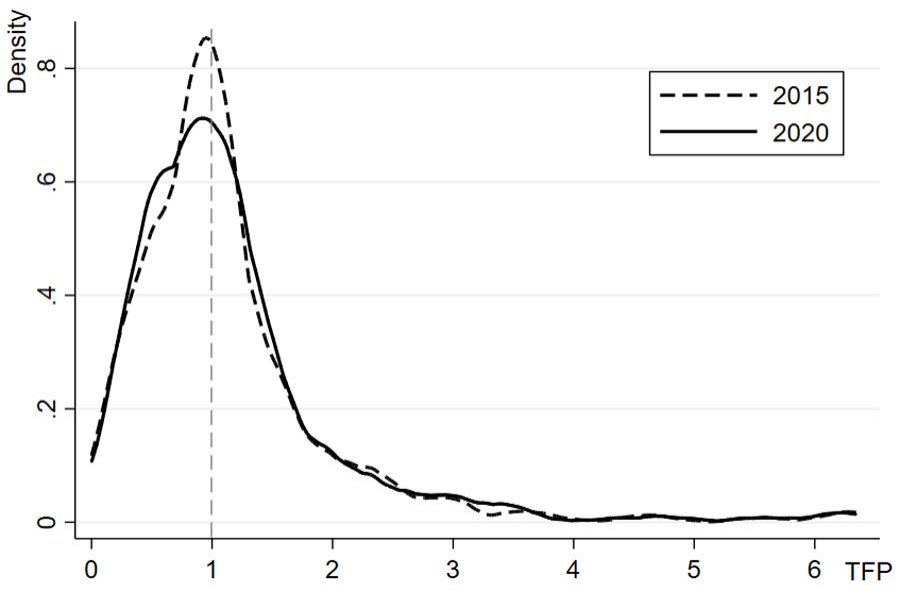
Kernel Density Distribution of Herdsmen’s Grass and Animal Husbandry Production Efficiency.

**Fig 2:**
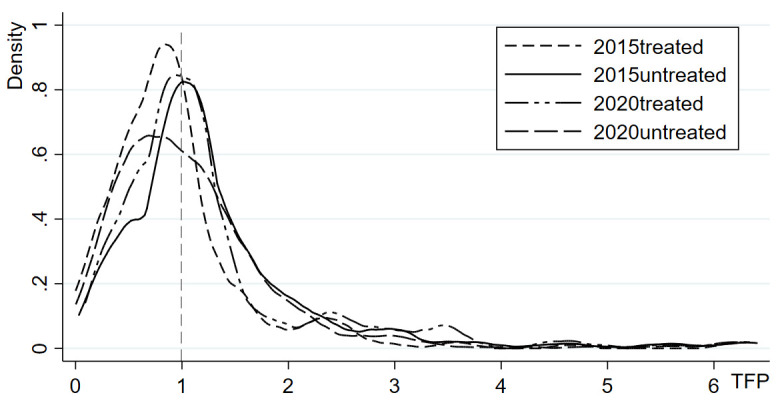
Kernel Density Distribution of Herdsmen’s Grass and Animal Husbandry Production Efficiency.

This study employs the DEA-Malmquist index method to measure total factor productivity (TFP) with 2010 as the base period during the implementation of the compensation policy, thereby representing herdsmen’s grass and animal husbandry production efficiency. Table 5 presents the production efficiency and its decomposition during the first and second policy phases. Overall, the grass and animal husbandry production efficiency of herdsmen showed a pattern of initial decline followed by recovery from 2010 to 2020. During 2011-2015, the TFP decreased by an average of 11.3% compared to 2010, primarily driven by a -20.9% average growth rate in technological progress, which was the main contributor to the negative growth in production efficiency. Although TFP in the 2016-2020 period increased by 0.98% compared to the first policy phase, it remained below the pre-policy implementation level overall. Furthermore, the distribution of herdsmen with TFP>1 showed greater dispersion compared to those with TFP<1 (Fig 1). Further analysis comparing participating and non-participating herdsmen reveals distinct patterns: while the control group showed continuous decline in TFP, the treatment group demonstrated sustained improvement from 2010 to 2020. During the first policy phase, participating herdsmen achieved an average 5.3% increase in production efficiency compared to 2010, mainly driven by simultaneous improvements in pure technical efficiency (1.171) and scale efficiency (1.089). In the second policy phase, TFP among participating herdsmen continued to rise to 1.145, accompanied by the highest level of comprehensive technical efficiency change index, which increased by 84.5% and served as the primary driver for TFP growth. However, technological progress consistently exerted a downward pressure on TFP throughout both periods. The kernel density distribution (Fig 2) indicates that the peaks of TFP curves for policy participants in both phases shifted upward compared to non-participants, with reduced curve width, suggesting more concentrated efficiency distributions among participating herdsmen and a converging trend in production efficiency disparities, indicating dynamic convergence characteristics. These findings reveal the general level and structural features of production efficiency among sample herdsmen. This leads to a more fundamental research question: how does participation in the Grassland Ecological Compensation Policy affect this efficiency? The subsequent section will employ econometric models to conduct rigorous causal inference analysis on this relationship.

**Table 5:** Total factor productivity and its decomposition in herdsmen’s grass and animal husbandry.

Period	Group	Comprehensive Technical Efficiency	Technological Progress	Pure Technical Efficiency	Scale Efficiency	Total Factor Productivity
**First Policy Period**	Overall	1.122	0.791	1.009	1.112	0.887
	Treatment Group	1.275	0.826	1.171	1.089	1.053
	Control Group	1.037	0.776	0.923	1.123	0.804
**Second Policy Period**	Overall	1.654	0.543	1.446	1.144	0.897
	Treatment Group	2.121	0.540	1.788	1.186	1.145
	Control Group	1.412	0.544	1.263	1.118	0.768

### Analysis of the impact of policy participation behavior on the production efficiency of herdsmen’s grass husbandry

#### Common support domain and balance test.

**Fig 3:**
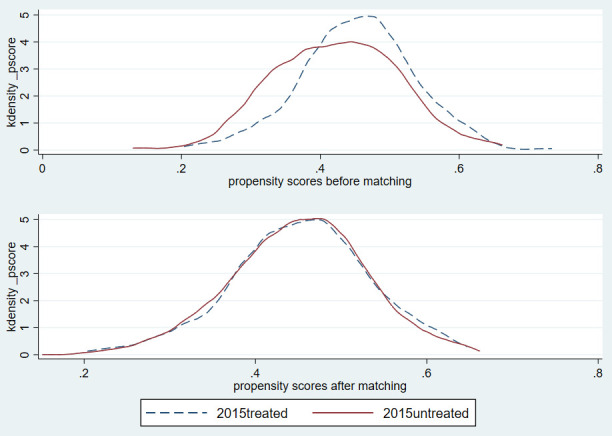
Kernel density function before and after propensity score matching during the first round of policies.

**Fig 4:**
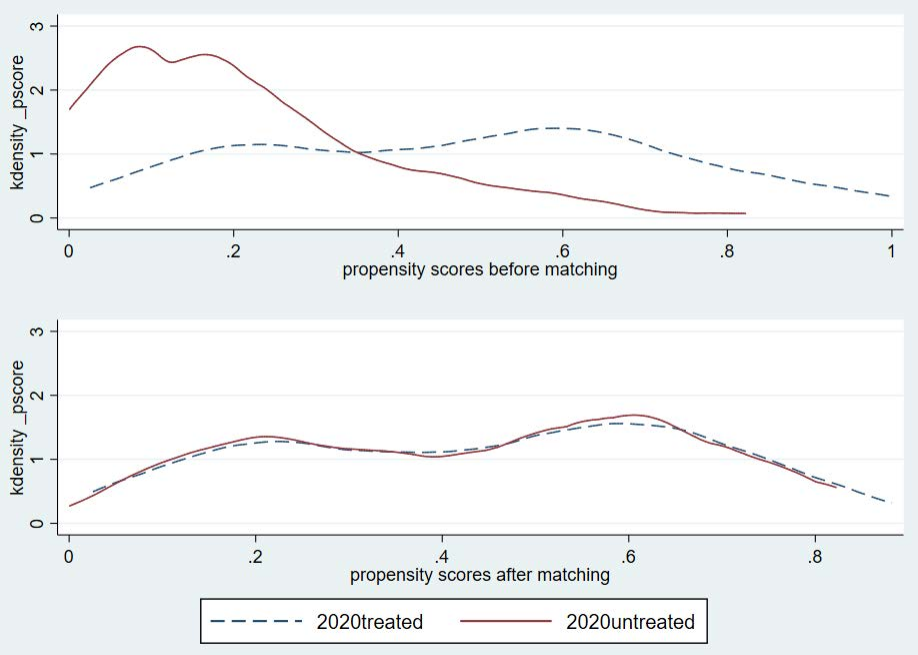
Kernel density function before and after propensity score matching during the second round of policies.

This paper uses the propensity score matching method to analyze whether there is a significant difference in the impact of herdsmen’s policy participation behavior on their grass and animal husbandry production efficiency during the first round and the second round of supplementary award policy. Table 6 reports the impact of the control variables using the logistic model on the policy participation behavior of herdsmen. From the coefficient estimation results, it can be concluded that whether farmers are part-time employed, travel convenience, compensation amount, yearly consumption expenditure, whether they are fined, the degree of supervision over herdsmen and some regions will have a significant impact on the policy participation behavior of herdsmen during the first or second round of policies. In order to ensure the quality of matching, after obtaining the propensity score of herdsmen’s policy participation behavior, it is necessary to further discuss the common support domain conditions of matching [26]. Fig 3 and Figure 4 shows the tendency score nuclear density curve before and after PSM. It can be seen that the propensity score ranges of the treatment group and the control group overlap in a considerable range during the first and second round of policies after matching, and the common support domains are [0.230, 0.609] and [0.081, 0.987] respectively. The sample herdsmen can achieve effective matching and meet the conditions of the common support domain. In addition, the nuclear density function diagram can also be used to test the quality of PSM, that is, the more overlapping parts of the nuclear density diagram of the treatment group and the control group, the better the matching effect [27]. Figs 3 and 4 shows that before PSM, the skewness and kurtosis of the nuclear density curve of the control group during the first and second round of policies deviated greatly from that of the treatment group. After PSM, the nuclear density curves of the control group and the treatment group were basically consistent. This shows that the comprehensive characteristics of herdsmen in the control group are similar to those in the treatment group, and the quality of propensity matching is better.

**Table 6:** Logistic regression results of propensity score.

Variable	2015		2020	
	Regression coefficient.	Standard error	Regression coefficient.	Standard error
**Gender**	−0.170	0.190	0.124	0.433
**Age**	−0.002	0.009	−0.000	0.011
**Education level**	−0.124^*^	0.083	−0.054	0.099
**Whether to work part-time**	0.288	0.194^*^	0.271	0.235
**Family dependency ratio**	−0.176	0.380	−0.224	0.472
**Distance to the market**	−0.0001	0.001	−0.003	0.004
**Travel convenience**	0.259	0.194^*^	−0.204	0.246
**Compensation amount**	−0.167	0.097^*^	0.467^***^	0.069
**Yearly consumption expenditure**	0.008	0.028	−0.071^**^	0.038
**Whether to be fined**	−0.348	0.217^*^	−0.689^**^	0.247
**Government supervision strength**	0.178	0.094^**^	−0.212^*^	0.132
**Regins**				
**Xilin Gol**	0.070	0.287	0.821^**^	0.323
**Chifeng**	−0.320	0.290	−2.334^***^	0.506
**Tongliao**	−0.093	0.303	0.293	0.393
**Ulanqab**	0.243	0.295	−0.369	0.407
**Constant term**	−0.083	0.618	−0.621	0.913

*, * *, * * * are significant at the level of 10%, 5% and 1% respectively, the same below.

**Fig 5:**
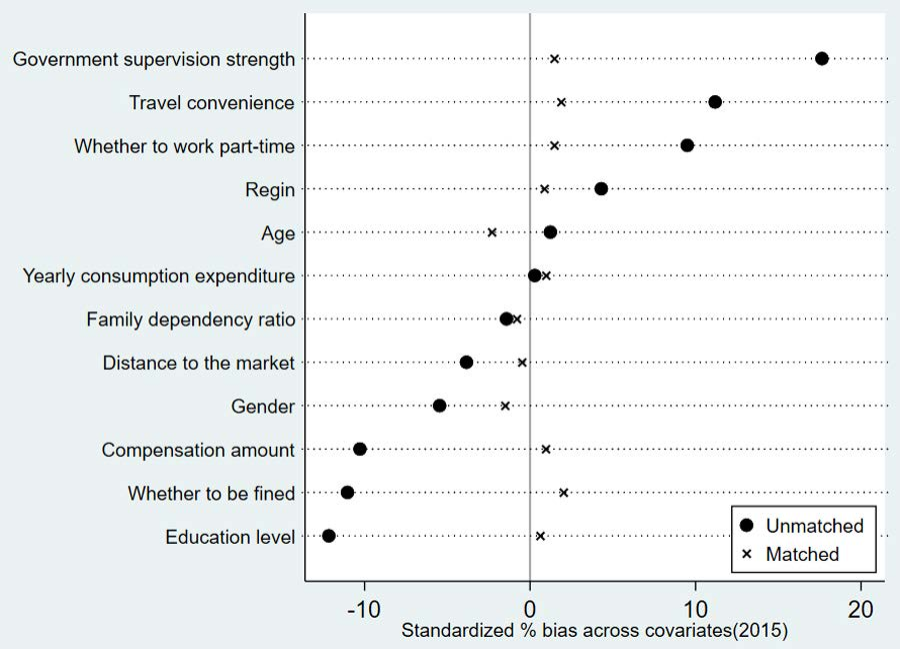
Standardized % bias across covariates during the first round of policies.

**Fig 6:**
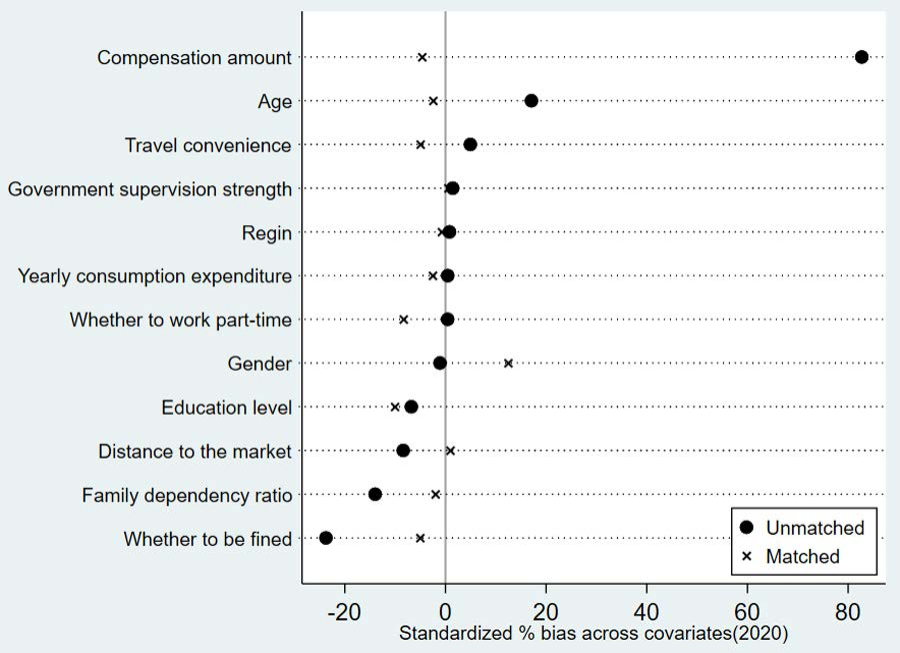
Standardized % bias across covariates during the second round of policies.

In order to ensure the reliability of the estimation results of ATT effect of herdsmen’s policy participation behavior on their grass and animal husbandry production efficiency during the first and second round of grassland ecological compensation policy by using PSM. Balance test is required to determine whether the absolute value of standard deviation of matched variables after PSM is less than 20% [28], so as to check whether there is no significant difference between the control group and the treatment group in each matched variable [25]. Two methods of kernel matching and neighbor matching are used for PSM analysis. Because the results of kernel matching and neighbor matching are similar, only the results of kernel matching balance test are listed in this paper. The results in Table 7, Fig 5 and 6 show that after PSM, the absolute value of the standard deviation of the matching variables of the first round and the second round of policy participation behavior is within 15%. And from the t-test results, it can be seen that the individual differences of the common control variables that affect the herdsmen’s policy participation behavior after matching between the treatment group and the control group are basically eliminated, and are no longer significant. This shows that the study has passed the balance test, and the estimation result of PSM is reliable and effective.

**Table 7:** Balance test results of matched variables.

Variable	Before and after matching	2015					2020				
		Treated group	Unreated group	Standard deviation%	Decrease (%)	T value	Treated group	Unreated group	Standard deviation%	Decrease (%)	T value
**Gender**	U	0.73	0.75	−5.5	47.4	−0.68	0.93	0.93	−1.1	−888.7	−0.12
	M	0.73	0.74	−2.9		−0.33	0.93	0.90	10.7		0.94
**Age**	U	43.52	43.39	1.2	91.6	0.15	49.09	47.25	17.1	73.4	1.97**
	M	43.52	43.53	−0.1		−0.01	49.09	48.60	4.5		0.43
**Education level**	U	2.07	2.20	−12.2	74.6	−1.69*	2.09	2.16	−6.8	−68.7	−0.78
	M	2.07	2.10	−3.1		−0.38	2.09	2.22	−11.4		−1.04
**Whether to work part-time**	U	0.27	0.23	9.5	98.2	2.18**	0.29	0.28	0.4	−1916.9	0.05
	M	0.27	0.27	−0.2		−0.02	0.29	0.33	−8.7		−0.82
**Family dependency ratio**	U	0.39	0.39	−1.4	−284.6	−0.18	0.36	0.40	−14	60.8	−1.73*
	M	0.39	0.40	−5.5		−0.64	0.36	0.38	−5.5		−0.52
**Distance to the market**	U	52.79	57.42	−3.8	99.8	−0.5	24.70	28.95	−8.4	63.9	−0.84
	M	52.79	52.80	0		0	24.70	23.17	3		0.56
**Travel convenience**	U	0.70	0.65	11.2	71.7	1.68*	0.69	0.66	4.9	−127.4	0.56
	M	0.70	0.69	3.2		0.37	0.69	0.74	−11.2		−1.11
**Compensation amount**	U	1.00	1.10	−10.3	16.9	−1.24	3.68	1.62	82.7	84.6	10.76***
	M	1.00	0.91	8.5		1.21	3.68	3.36	12.7		1.11
**Yearly consumption expenditure**	U	4.24	4.23	0.3	−524.8	0.04	4.48	4.46	0.5	−1718.7	0.05
	M	4.24	4.18	1.8		0.21	4.48	4.19	8.2		0.82
**Whether to be fined**	U	0.28	0.33	−11	89.8	−1.96**	0.30	0.41	−23.7	93.2	−2.65***
	M	0.28	0.28	1.1		0.13	0.30	0.29	1.6		0.16
**Government supervision strength**	U	2.28	2.11	17.6	95.6	2.17**	2.52	2.50	1.5	−152.6	0.16
	M	2.28	2.29	−0.8		−0.09	2.52	2.48	3.7		0.37
Regin	U	2.83	2.77	4.3	85.1	0.53	2.81	2.80	0.8	−383.9	0.09
	M	2.83	2.82	0.6		0.07	2.81	2.75	3.9		0.35

#### Analysis on ATT effect of herdsmen’s grass and animal husbandry production efficiency.

This paper uses the propensity score matching method to analyze the ATT effect of herdsmen’s policy participation behavior on their grass and animal husbandry production efficiency during the first and second round of grassland ecological compensation policy. In order to ensure the accuracy of the impact effect of grassland ecological compensation policy, this paper uses two matching methods, kernel matching method and nearest neighbor matching method, to test the average processing effect of herdsmen’s grass and animal husbandry production efficiency. The results are shown in Table 8. During the first round of policy implementation, the production efficiency of herdsmen’s grass husbandry who did not participate in the policy was significantly higher than those who participated in the policy. The results of kernel matching and neighbor matching showed that the average production efficiency of herdsmen in the treatment group was 1.069 and 1.071, respectively, while the average production efficiency of herdsmen in the control group was 1.450 and 1.322, respectively. The ATT of treatment effect was −0.381 and −0.251, respectively, which passed the significance test of 5%. It shows that the policy participation behavior of herdsmen during the first round of policy did not significantly improve their grass and animal husbandry production efficiency, but was lower than that of herdsmen who did not participate.

**Table 8:** ATT effect of herdsmen’s policy participation behavior on grass and animal husbandry production efficiency.

Matching method		Treated group	Unreated group	ATT value	Standard Error	T value
**During the first round of grassland ecological subsidy policy**	kernel matching	1.069	1.450	−0.381	0.154	−2.42^**^
	neighbor matching (1–5)	1.071	1.322	−0.251	0.120	−2.08^**^
	Mean	1.070	1.386	−0.316	–	
**During the second round of grassland ecological subsidy policy**	kernel matching	1.213	1.136	0.076	0.144	0.53
	neighbor matching (1–5)	1.198	1.081	0.117	0.157	0.75
	Mean	1.206	1.109	0.097	–	–

Compared with the ATT effect of herdsmen’s grass and animal husbandry production efficiency during the first round of policy, during the second round of policy implementation, the grass and animal husbandry production efficiency of herdsmen who participated in the policy was higher than that of herdsmen who did not participate. The average production efficiency of herdsmen with policy participation behavior in the kernel matching method was 1.213, and the average production efficiency of herdsmen without participation was 1.136. The average production efficiency of herdsmen with policy participation behavior in the nearest neighbor matching method was 1.198, while the average production efficiency of herdsmen without reducing livestock to the livestock carrying capacity specified in the policy was 1.081. ATT values were 0.076 and 0.117, respectively, indicating that the policy participation behavior of herdsmen during the second round of policy improved their grass and animal husbandry production efficiency. Although the results are not significant, it can be seen from the comparison that with the continuous promotion and optimization of policies, the impact of herdsmen’s policy participation behavior on their grass and animal husbandry production efficiency has shifted from negative inhibition to positive promotion.

### Threshold effect analysis: herdsmen’s perception of threshold effect

#### Multicollinearity test of perception variables.

To ensure the robustness of the model results, this study conducted multicollinearity diagnostics on the five perception dimension variables (economic perception, loss perception, ecological perception, emotional perception, and overall perception). Since these variables are incorporated into the panel threshold model as threshold variables separately rather than simultaneously as regressors, traditional Variance Inflation Factor (VIF) testing is not entirely applicable. Nevertheless, to gain deeper insights into the intrinsic relationships among the variables, Pearson correlation analysis was employed for examination.

The test results, presented in Table 9, reveal several key findings. First, the pairwise correlation coefficients among the four specific perception dimensions (economic, loss, ecological, and emotional) are all below 0.3, with the highest being 0.4826. This indicates that these four variables exhibit good statistical independence, measuring distinct aspects of herdsmen’s subjective perceptions, thereby justifying their separate examination for threshold effects in this study. Second, moderate to strong correlations (ranging from 0.5043 to 0.7290) are observed between overall perception and each specific dimension. This aligns with theoretical expectations, validating that “overall perception” serves as a comprehensive indicator derived from the aggregation of specific dimensional perceptions, reflecting the internal consistency of the questionnaire design. All correlation coefficients remain below the critical threshold of 0.8, which would indicate severe multicollinearity. Therefore, the perception variable data in this study do not exhibit serious multicollinearity issues and are suitable for subsequent econometric analysis.

**Table 9:** Multicollinearity test of perception variables.

Variable	Economic perception	Loss perception	Ecological perception	Emotional perception	Overall perception of herdsmen
**Economic perception**	1				
**Loss perception**	0.1605	1			
**Ecological perception**	0.4826	0.1245	1		
**Emotional perception**	0.2973	0.194	0.2882	1	
**Overall perception of herdsmen**	0.7186	0.5043	0.7	0.729	1

#### Test of threshold variables.

In this paper, the overall perception, economic perception, loss perception, emotional perception and Ecological Perception of herdsmen are set as threshold variables into the model, and the panel threshold model is processed based on stata15.0 software. The relationship between policy participation behavior, herdsmen’s perception and their grass and animal husbandry production efficiency was analyzed. The significance and threshold value of the threshold number of the model are tested according to the “bootstrap method”, and the test results are shown in Table 10. The results demonstrate that the impact of herders’ policy participation behavior on grass and animal husbandry production efficiency exhibits varying degrees of single- and double-threshold effects. Both single- and double-threshold effects for overall herdsman perception, economic perception, and loss perception are statistically significant at the 10% level. When ecological perception and emotional perception serve as threshold variables, the regression models show significant single-threshold effects but no significant double-threshold effects. Therefore, this paper will use the double threshold panel model based on Herdsmen’s overall perception level, herdsmen’s economic perception and loss perception, and the single threshold panel model based on Ecological Perception and emotional perception for empirical analysis.

**Table 10:** Effect test of Threshold Panel Model.

dependent variable	Threshold variable	Single threshold test					Double threshold test				
		F value	P value	1%	5%	10%	F value	P value	1%	5%	10%
**Production efficiency of animal husbandry**	**Herdsman perception**	19.950	0.073	18.531	24.493	47.449	13.980	0.093	13.714	19.950	0.073
	**Economic perception**	16.410	0.040	10.351	14.176	26.638	13.860	0.080	12.885	16.410	0.040
	**Loss perception**	14.330	0.077	11.922	17.820	22.742	12.980	0.099	12.835	14.330	0.077
	**Ecological perception**	14.530	0.080	13.412	18.475	35.958	3.690	0.370	12.854	14.530	0.080
	**Emotional perception**	14.960	0.070	12.641	16.295	39.127	2.660	0.460	11.454	14.960	0.070

#### Analysis of regression results.

Through the above analysis, it can be seen that there is a double threshold effect in the relationship between herdsmen’s family policy participation behavior and their grass and animal husbandry production efficiency, with the overall subjective perception of herdsmen as the threshold variable. The regression results in Table 11 show that there is a “U-shaped” relationship between herdsmen’s policy participation behavior and their grass and animal husbandry production efficiency, that is, the relationship between inhibition and promotion. When herdsmen perception levels fall below the threshold of 2.625, the regression coefficient reaches −1.753, indicating that policy participation leads to an approximately 175.3% decline in TFP, statistically significant at the 5% level. This finding suggests that when herdsmen hold strongly negative evaluations of the policy, mandatory participation not only fails to generate incentives but substantially dampens production motivation, resulting in a sharp deterioration of production efficiency. As perception levels rise to the medium range (2.625–3.625), the negative relationship moderates to a coefficient of −0.143. When perception reaches the high threshold bracket, the coefficient turns positive at 0.202, demonstrating a potentially beneficial effect. However, since the majority of herdsmen maintain relatively low perception levels concentrated in the lower threshold range, this positive impact of policy participation in the high perception bracket does not achieve statistical significance.

**Table 11:** Estimation results of threshold model parameters.

Threshold variable	Threshold interval	Regression coefficient	Standard error	T value	P value	F value
**Herdsman perception**	(perception_*it*_ < 2.625)	−1.753^***^	0.580	−3.020	0.003	2.24^***^
	(2.625 ≤ perception_*it*_ < 3.625)	0.143	0.340	−0.420	0.674	
	(perception _*it*_ ≥ 3.625)	0.202	0.338	0.600	0.551	
**Economic perception**	(economy_*it*_ < 2.5)	−0.917^*^	0.467	−1.960	0.051	3.92^***^
	(2.5 ≤ economy_*it*_ < 3)	0.195^*^	0.406	1.860	0.064	
	(economy_*it*_ ≥ 3)	0.332	0.312	1.070	0.287	
**Loss perception**	(perception_*it*_ < 2)	−0.824^*^	0.500	−1.670	0.095	3.75^***^
	(2 ≤ perception_*it*_ < 3.5)	0.026	0.339	0.180	0.858	
	(perception_*it*_ ≥ 3.5)	0.301	0.324	0.960	0.339	
**Ecological perception**	(ecology_*it*_ < 3)	−0.768 ^**^	0.475	−2.450	0.015	4.43^***^
	(ecology_*it*_ ≥ 3)	0.171	0.291	0.590	0.556	
**Emotional perception**	(emotion_*it*_ < 3)	−1.056^**^	0.434	−2.430	0.016	2.21^**^
	(emotion_*it*_ ≥ 3)	0.053	0.302	0.170	0.862	

Economic perception and loss perception as threshold variables are similar to the overall perception level. There is a double threshold effect with herdsmen’s economic perception and loss perception as threshold variables, and it presents a “U-shaped” shape. This shows that under the stage of different degrees of economic perception and loss perception, the impact of herdsmen’s policy participation behavior on their grass and animal husbandry production efficiency is first negative inhibition, and then positive promotion. When economic perception falls below the threshold of 2.5, the regression coefficient is significantly negative at −0.917. As herders’ economic perception crosses the first threshold (2.5) but remains below the second threshold (3.0), the marginal effect of policy participation on Total Factor Productivity (TFP) turns positive, with a coefficient of 0.195 (significant at the 10% level). This indicates that within this perception range, policy participation leads to an approximately 19.5% improvement in TFP. The findings suggest that when compensation policies enable herders to perceive tangible economic benefits – reaching what might be termed a ‘subsistence line’ though not yet a ‘prosperity line’ – they begin to adjust production strategies. Consequently, policy participation transforms from a ‘livelihood burden’ into an opportunity for efficiency enhancement, significantly improving resource allocation efficiency. Currently, the majority of herders have surpassed the lower threshold of economic perception, indicating that policy participation can effectively promote production efficiency in grass and animal husbandry. Furthermore, when economic perception reaches the higher threshold stage, it can achieve an increasingly positive promotional effect between these two factors. This threshold value demonstrates clear real-world correspondence through field interviews. Herders with economic perception below this threshold commonly expressed that “compensation cannot offset the income from raising fewer sheep,” exhibiting passive compliance in their behavior. For instance, Herder A stated: “The policy requires livestock reduction, so I sold some animals. But I haven’t considered how to make the remaining sheep more profitable - the compensation is just enough to get by.” Production adjustments under this mindset tend to be mechanical and unlikely to improve efficiency. In contrast, herders exceeding this threshold begin to view compensation policy as ‘start-up capital’ or ‘risk protection,’ actively optimizing their production. As Herder B explained: “I’ve done the calculations - relying solely on compensation and traditional grazing isn’t sufficient. I used this money to purchase high-quality breeding sheep. Although the number of animals decreased, the faster turnover and premium prices actually increased my total income.” This indicates that the threshold essentially marks a crucial psychological turning point where herders transition from ‘passive acceptance of compensation’ to ‘active pursuit of transformation,’ consequently upgrading their production behavior from simple livestock reduction to comprehensive optimization, thereby driving substantial improvements in production efficiency.

Regarding loss perception, when the measurement degree falls below 2, policy participation significantly inhibits production efficiency in grass and animal husbandry (approximately 82.4% decrease). Under these conditions, policy participation fails to enhance production input-output efficiency and instead creates obstacles. This may stem from a ‘passive acceptance’ mentality: while herders may not perceive enormous losses, they similarly lack motivation to actively pursue production optimization within policy constraints. When loss ≥ 2, there is an upward positive correlation between the two. At present, the overall loss perception of herdsmen is low, so the positive role of herdsmen ‘policy participation behavior in the high threshold stage is not significant. Comparing the threshold range of economic perception and loss perception, it can be seen that when the relationship between herdsmen’s policy participation behavior and their grass and animal husbandry production efficiency turns from negative to positive, the threshold value of loss perception is lower than economic perception, indicating that herdsmen have “loss aversion” behavior [29]. Herdsmen are more sensitive to known losses than to unknown gains.

In addition, there is a single threshold effect of herdsmen’s Ecological Perception and emotional perception as threshold variables in the impact of herdsmen’s family policy participation on their grass and animal husbandry production efficiency, showing a “U-shaped” shape of first inhibition and then promotion. When ecological and emotional perception remain at low threshold levels (below 3.0), the regression coefficients between herders’ policy participation and production efficiency stand at −0.768 and −1.056 respectively. This indicates a significant negative relationship, where policy participation leads to 76.8% and 105.6% decreases in production efficiency, both statistically significant at the 5% level. Once these perception levels cross the threshold (≥3.0), the negative impact of policy participation is eliminated, with coefficients turning positive (17.1% and 5.3% respectively), though not statistically significant. The results demonstrate that ecological and emotional perception serve as “stabilizers” enabling herders to accept policies and endure transitional challenges in production transformation. Only when herders genuinely identify with the policy’s ecological value and develop a sense of belonging will they willingly tolerate short-term efficiency losses and make adjustments for long-term sustainable development. Without such identification, policy participation directly translates into production efficiency losses. Field investigations reveal that herders with low ecological and emotional perception predominantly view the policy as an external constraint, whereas those with high perception levels demonstrate strong endogenous conservation motivation. For instance, Herder C expressed: “When the grassland was degraded, I felt anxious. Now with the vegetation recovering, I feel secure. Raising fewer animals but managing them better represents a sustainable long-term strategy.” This cognitive framework, which deeply intertwines personal interests with grassland health, transcends short-term “economic calculation” and embraces long-term “eco-economic coordination.” Consequently, after crossing the perception threshold, herders show greater willingness to adopt practices that entail higher short-term costs but deliver long-term benefits. These behavioral adaptations establish a solid foundation for sustainable productivity improvement over the long term.

## Discussion

This paper takes the relationship between herdsmen’s policy participation behavior and grass husbandry production efficiency under the grassland ecological compensation policy as the breakthrough point, and empirically studies the difference between grass husbandry production efficiency and herdsmen’s policy participation behavior. This is of great practical significance to guide and encourage herdsmen ‘families to protect the grassland ecological environment, optimize the grassland ecological subsidy policy, and help the Rural Revitalization Strategy.

From the impact of policy participation behavior on the production efficiency of herdsmen’s grass and animal husbandry, it can be seen that with the continuous promotion and optimization of grassland ecological compensation policy, the relationship between herdsmen’s policy participation behavior and their grass and animal husbandry production efficiency has gradually changed from negative inhibition to positive promotion. It can be seen that there is a short-term trade-off and long-term synergy between the policy participation behavior of herdsmen in implementing the subsidy policy and their grass and animal husbandry production. Therefore, in the process of policy implementation, the government should continue to optimize the subsidy policy, mobilize the enthusiasm of herdsmen to protect the grassland ecological environment, and guide and encourage herdsmen ‘families to actively participate in the grassland ecological subsidy policy. While protecting grassland ecology, we should realize the sustainable improvement of grass and animal husbandry production.

Furthermore, logistic regression estimates reveal that part-time employment significantly positively influenced herders’ policy participation during both policy phases. Therefore, policymakers should emphasize encouraging diversified livelihoods among herders. By establishing an income diversification support system that incentivizes part-time employment, stakeholders can stabilize income sources and enhance herders’ economic standing. This approach can effectively moderate the relationship between livestock reduction and production efficiency while facilitating the transition of herder households’ production models. Policy should proactively shift from “tacit permission of diversification” to “active encouragement and empowerment of diversification,” systematically supporting herders in developing multiple income channels to reduce over-reliance on grass-based animal husbandry revenue. Specific measures include: (1) Cultivating new formats in grassland tourism and ecological services by encouraging and supporting herders to leverage unique grassland landscapes, ethnic culture, and family ranch resources to develop cultural tourism services – including small-scale family homestays, immersive grassland experiences, and nature education programs – while maintaining grass-livestock balance. (2) Comprehensively promoting the processing of characteristic livestock products and value chain extension through support for herder cooperatives and local small enterprises to engage in primary and deep processing of green and organic animal products. (3) Actively exploring innovative pathways to transform grassland ecological conservation achievements into economic benefits, creating new mechanisms for translating environmental protection efforts into tangible financial returns for local communities.

There is a “U-shaped” double threshold effect between herdsmen’s perception of their policy participation behavior and the production efficiency of grassland and animal husbandry, with herdsmen’s overall perception, economic perception and loss perception as threshold variables. And the “U-shaped” single threshold effect with Ecological Perception and emotional perception as threshold variables. From the empirical results, it can be seen that the economic perception of herdsmen has broken through the low threshold price, and the policy participation behavior of herdsmen can effectively promote the improvement of the production efficiency of their grass and animal husbandry. The overall perception, loss perception, ecological perception and emotional perception of herdsmen are still in the low threshold range, and the positive role of herdsmen’s family policy participation behavior in the high threshold stage is not significant. The promotion effect of herdsmen’s subjective perception on the relationship between policy participation behavior and grass and animal husbandry production efficiency has not been effectively played. In addition, compared with the threshold value of the negative to positive relationship between herdsmen’s policy participation behavior and their grass husbandry production efficiency, loss perception<economic perception<overall perception<Ecological Perception = emotional perception. It can be seen that, compared with the positive economic perception caused by the reduction of livestock, the loss caused by the herdsmen according to the policy and the loss perception of the costs invested are more sensitive to the regulation of the relationship between the policy participation behavior and the production efficiency of grassland and animal husbandry. Therefore, in the process of specific optimization policies, the government can appropriately improve the compensation standard in combination with regional differences. The uniform compensation standard fails to achieve the policy objective of coordinating ecological protection with herders’ livelihoods. Future policy optimization must transition from “universal compensation” to “targeted incentives.” Based on our findings, we propose a three-dimensional differentiated compensation model incorporating “grassland type - stocking rate - livestock reduction intensity.” This model establishes a sophisticated compensation framework that accounts for key ecological and behavioral factors: (1) Compensation standards should prioritize grassland types with higher ecological vulnerability, lower restoration capacity, and greater ecological significance (e.g., desert steppes), reflecting the “ecological cost” dimension. (2) Using pre-policy initial stocking rates as baseline, the model distinguishes historical utilization intensity. Herders who previously maintained stocking rates within sustainable limits deserve premium rewards for additional conservation efforts, ensuring fairness and avoiding penalizing responsible practitioners. (3) Greater livestock reduction intensity warrants higher compensation, as it represents more substantial ecological contribution and livelihood sacrifice. This dimension directly correlates with herders’ economic perception and serves as a crucial incentive for policy engagement. The three-dimensional model internalizes ecological externalities through differentiated institutional design, effectively guiding herder behavior toward promoting both ecological and economic sustainability in grassland regions.

Concurrently, government agencies should enhance policy communication efforts to help herders better understand the ecological and economic benefits derived from regulated livestock reduction. Such initiatives would improve herders’ ecological and emotional perception, fostering grassroots awareness of grassland conservation and motivating sustainable practices in grass-based animal husbandry.

## Conclusions

Based on 536 field survey data of herdsmen in Inner Mongolia, the herdsmen with policy participation behavior were selected as the treatment group, and the herdsmen without policy participation were selected as the control group. The propensity score matching method was used to analyze whether there was a significant difference in the impact of herdsmen’s policy participation behavior on their grass and animal husbandry production efficiency during the first and second rounds of policies. On this basis, the threshold effect of herdsmen’s participation in policies on their grass and animal husbandry production efficiency was further analyzed with herdsmen’s perception as the threshold variable, and the following conclusions were obtained.

(1) ATT effect of herdsmen’s policy participation behavior on grass and animal husbandry production efficiency shows that the policy participation behavior of herdsmen during the first round of policy is not conducive to the improvement of grass and animal husbandry production efficiency. During the second round of the policy, the production efficiency of herdsmen’s grass and animal husbandry who reduced livestock to the carrying capacity specified in the policy was higher than that of herdsmen’s grass and animal husbandry who did not reduce livestock. The analysis reveals that as the Grassland Ecological Compensation Policy progressed, its impact on production efficiency in grass and animal husbandry transitioned from negative to positive. The initial policy phase exhibited dual challenges of “rigid constraints” and “insufficient compensation.” During this exploratory stage, the top-down approach to rapid livestock reduction failed to provide adequate adjustment periods for production restructuring. Consequently, fixed assets (such as pens and equipment) and labor inputs could not be adjusted synchronously with the sharp decline in livestock inventory. Simultaneously, the compensation standards during the initial phase inadequately covered the substantially increased variable costs resulting from purchasing supplemental feed to replace natural grazing. This created an operational dilemma where herders faced declining income alongside rising costs, manifesting as temporary suppression of production efficiency in the data. From the perspective of behavioral adaptation, herders’ decision-making objectives shifted from long-term profit maximization to short-term risk minimization when confronting significant policy uncertainty. This behavioral response inevitably led to increased short-term production costs and efficiency losses in resource allocation. In conclusion, the productivity suppression observed during the first policy phase represents transitional challenges in policy implementation. These findings underscore that achieving ecological-economic synergies in ecological compensation policies requires careful consideration of policy gradualism, compensation adequacy, and complementary technical training and industrial guidance. Such comprehensive approaches can effectively support herders through production transition periods, providing clear directions for optimizing subsequent policy phases.

(2) The threshold effect of herdsmen’s perception shows that there is a “U-shaped” double threshold effect between herdsmen’s policy participation behavior and their grass and animal husbandry production efficiency, with the overall perception of herdsmen as the threshold variable. The threshold value of the regression coefficient from negative correlation to positive correlation is 3.625. Because the overall perception level of herdsmen is low, the positive role of herdsmen’s policy participation behavior in the high threshold stage is not significant. Economic perception and loss perception as threshold variables are similar to the overall perception level. Economic perception shows a significant positive relationship in the threshold range of [2.5, 3), and the positive promotion effect of herdsmen’s policy participation behavior in the high threshold stage of loss perception is not significant. The impact of herdsmen’s policy participation behavior on their grass and animal husbandry production efficiency has a single threshold effect with herdsmen’s Ecological Perception and emotional perception as threshold variables, showing a “U-shaped” shape of first inhibition and then promotion. Both ecological perception and emotional perception show a significant negative relationship in the threshold range of [1, 3).
